# Use of an Animal Model to Evaluate Anxiolytic Effects of Dietary Supplementation with *Tilia tomentosa* Moench Bud Extracts

**DOI:** 10.3390/nu12113328

**Published:** 2020-10-29

**Authors:** Federica Turrini, Giulia Vallarino, Francesca Cisani, Dario Donno, Gabriele Loris Beccaro, Paola Zunin, Raffaella Boggia, Anna Pittaluga, Massimo Grilli

**Affiliations:** 1 Department of Pharmacy, University of Genoa, Viale Cembrano 4, 16148 Genoa, Italy; turrini@difar.unige.it (F.T.); vallarino@difar.unige.it (G.V.); cisani@difar.unige.it (F.C.); zunin@difar.unige.it (P.Z.); boggia@difar.unige.it (R.B.); pittalug@difar.unige.it (A.P.); 2 Department of Agriculture, Forestry and Food Science, University of Torino, Largo Braccini 2, 10095 Grugliasco (TO), Italy; dario.donno@unito.it (D.D.); gabriele.beccaro@unito.it (G.L.B.)

**Keywords:** polyphenols, bud-derivatives, nutraceuticals, supplements, anxiety, mood disorders, mice behaviour, principal component analysis

## Abstract

Anxiety disorders are common and complex psychiatric syndromes affecting a broad spectrum of patients. On top of that, we know that aging produces an increase in anxiety vulnerability and sedative consumption. Moreover, stress disorders frequently show a clear gender susceptibility. Currently, the approved pharmacological strategies have severe side effects such as hallucinations, addiction, suicide, insomnia, and loss of motor coordination. Dietary integration with supplements represents an intriguing strategy for improving the efficacy and the safety of synthetic anxiolytics. Accordingly, a recent article demonstrated that glyceric bud extracts from *Tilia tomentosa* Moench (TTBEs) exert effects that are consistent with anxiolytic activity. However, the effects of these compounds in vivo are unknown. To examine this question, we conducted behavioral analysis in mice. A total of 21 days of oral supplements (vehicle and TTBEs) were assessed by Light Dark and Hole Board tests in male and female mice (young, 3 months; old, 24 months). Interestingly, the principal component analysis revealed gender and age-specific behavioral modulations. Moreover, the diet integration with the botanicals did not modify the body weight gain and the daily intake of water. Our results support the use of TTBEs as dietary supplements for anxiolytic purposes and unveil age and gender-dependent responses.

## 1. Introduction

Anxiety and stress-related disorders are psychiatric conditions vulnerable to the influence of altered signaling from the gut microbiota [1]. Accordingly, the scientific community suggests that a correct interpretation of the diet and, if necessary, a targeted food supplementation can improve the effectiveness of therapies [2]. Another critical point in the management of anxiety-like disorders is the gender and age susceptibility [3,4,5,6]. Reporting data demonstrated that females are more affected by anxiety, and that, currently, both young and old people make wide use of anxiolytic drugs [7,8]. The social impact of anxiolytics consumption generates the need to strongly reduce the power of side effects [9,10,11]. Consequently, the integration of actual therapy with dietary supplements like botanicals could be recommended in the most fragile patients [12]. Interestingly, bud-derivatives, obtained by macerating meristematic fresh tissues of trees and herbaceous plants, represent a relatively new category of botanicals. In most countries of the EU, bud-derivatives, named also gemmoderivatives or embryo-extracts, are classified as plant food supplements [13,14,15,16,17]. The genotype of the considered species and varieties, the environmental characteristics of the sampling-sites, the phenological stage of the buds, the applied agrotechniques and the manufacturer practices influence the quality of these preparations [18].

*Tilia tomentosa* Moench buds are used worldwide for the preparation of tea and dietary supplements. This use is justified by a plethora of potential healthy properties attributed to *Tilia tomentosa* Moench Buds Extracts (TTBEs). TTBEs represent one of the dietary supplements investigated in the FINNOVER project (Innovative strategies for the development of cross-border green supply chains), a European Interreg Alcotra Italy/France project (2017–2020). Interestingly, TTTBEs are considered for their potential anxiolytic effects on the central nervous system (CNS) [19,20], probably due to their phenolic composition mainly represented by flavonols (quercetin, kaempferol, and apigenin derivatives) and phenolic acids. 

Recently, Allio and colleagues examined the impact of TTBEs at gamma-aminobutyric (GABA)ergic synapses by performing post-synaptic voltage-clamp recordings in neuronal cultures. Direct application of TTBEs on post-synaptic terminals activated a chloride current in a way consistent with the selective activation of GABA_A_ receptors [20]. Dysfunctions of the GABA system in the CNS have long been associated with anxiety and mood disorders [21,22]. Similarly, noradrenaline is also known to play a main role in the rapid responses to environmental stimuli and stress [23,24]. The GABAergic and the noradrenergic system are closely connected as demonstrated by reliable data in the literature showing GABA modulates the release of noradrenaline in the CNS. In particular, the GABAergic modulation involves presynaptic release-regulating GABAA receptors controlling noradrenaline exocytosis from noradrenergic nerve terminals [25,26]. Accordingly, the interplay between GABA and noradrenaline neurotransmission could explain the efficacy of pharmacological and/or nutraceutical approaches against mood illnesses. The hypothesis that TTBEs can influence this pathway also fully explains their effects when administered in vivo. 

Based on these observations, we decided to investigate whether dietary supplementation with TTBEs, marketed for human consumption, modify motor and behavioral skills in an animal model. Our results support the idea that dietary integration with TTBEs represent a nutritional strategy to counteract stress and anxiety symptoms also in human.

## 2. Materials and Methods 

### 2.1. Raw Samples

*Tilia tomentosa* Moench (Malvaceae) leaves were collected at embryonic stage as meristematic tissues: buds and young sprouts. The raw material was collected in the period February–April 2017 from plants spontaneously grown in the valleys of Chisone, Pellice, Germanasca, Bronda, and Varaita (Turin, Italy) and authenticated by a botanist. The manufacturing of the corresponding herbal preparations, Glyceric Macerates (GM), was performed by the encoded traditional method during 2017 in an Italian food supplements company (GEAL PHARMA—Turin, Italy). In detail, GM were prepared according to the European Pharmacopoeia 8th edition (Pharmaciens 1965), following the GM procedure deriving from the French Pharmacopoeia and adapted by the food supplements company using a cold maceration of the fresh raw material in a solution of water, 95% ethanol, and glycerol (50/20/30 *w*/*w*) with a 1:15 weight ratio between plant and solvent. The cold maceration process was protracted for 3 months, followed by a first filtration and, after 2 days of decanting, a second filtration (Whatman paper filter, n° 1, Sigma Aldrich, Milan, Italy). The obtained extracts (TTBEs), which represent the commercial products, were stored in dark bottles at normal atmosphere (N.A.), at 4 °C and 95% relative humidity until commercialization/use.

### 2.2. Spectroscopic Analysis: UV-Visible Fingerprint

UV–Visible absorption spectra (200–900 nm) were recorded by a spectrophotometer Agilent Cary 100 (Varian Co., Santa Clara, CO, USA) with 0.5 nm resolution, using rectangular quartz cuvettes with 1 cm path length. In order to avoid signal saturation GMs, before the spectroscopic analysis, were suitably diluted in the maceration solvent and thereafter spectra were acquired in duplicate and then averaged. The collection was performed at room temperature (25 ± 1 °C), against a blank solution represented by the dilution solvent. Standard normal variate (SNV) transform was later performed on the spectral data to remove or at least minimize any unwanted spectral contribution arising from multiplicative effects of scattering [27].

### 2.3. HPLC Analysis

In this study, fingerprint analysis for phytochemical characterization of samples were performed by different HPLC–DAD methods. Four polyphenolic classes were considered: benzoic acids (ellagic and gallic acids), catechins ((+)catechin and (−)epicatechin), cinnamic acids (caffeic, chlorogenic, coumaric, and ferulic acids), and flavonols (hyperoside, isoquercitrin, quercetin, quercitrin, and rutin). Total bioactive compound content (TBCC) was evaluated as the sum of the selected bioactive [27] compounds with health-promoting effects on human organism (“multimarker approach”) [28]. Biomarkers were selected for their demonstrated positive healthy properties and antioxidant capacity by literature in relation to the use of this plant-derived products.

The chromatographic analysis was performed by an Agilent 1200 High-Performance Liquid Chromatograph equipped with an Agilent UV-Vis diode array detector (Agilent Technologies, Santa Clara, CA, USA). Bioactive molecules were separated on a Kinetex C18 column (4.6 × 150 mm, 5 m, Phenomenex, Torrance, CA, USA). Several mobile phases were analyzed and UV spectra were recorded at different wavelengths, according to [10,18], with minor modifications: (i) a solution of 10 mM KH_2_PO_4_/H_3_PO_4_ (A) and acetonitrile (B) with a flow rate of 1.5 mL·min^−1^ (method A—analysis of cinnamic acids and flavonols, gradient analysis: 5% B to 21% B in 17 min + 21% B in 3 min + 2 min of conditioning time); (ii) a solution (A) of methanol/water/formic acid (5:95:0.1 *v*/*v*/*v*) and a mix (B) of methanol/formic acid (100:0.1 *v*/*v*) with a flow rate of 0.6 mL·min^−1^ (method B—analysis of benzoic acids and catechins, gradient analysis: 3% B to 85% B in 22 min + 85% B in 1 min + 2 min of conditioning time). UV spectra were recorded at 330 nm (A); 280 nm (B).

All single compounds were identified in samples by comparison and combination of their retention times and UV spectra with those of authentic standards in the same chromatographic conditions.

### 2.4. Animals

Male and female mice (strain C57BL/6J) were purchased from Charles River (Calco, Italy). Mice were reared up to 3 and 24 months in the animal facility of the Department of Pharmacy, Section of Pharmacology and Toxicology, School of Medical and Pharmaceutical Sciences, University of Genoa (authorization n. 484 of 8th June 2004). The experimental procedures have complied the European legislation (Directive 2010/63/EU for animal experiments), the ARRIVE guidelines, and they were approved by the Committee on the Ethics of Animal Experiments of the University of Genoa and by the Italian Ministry of Health (DDL 26/2014 and previous legislation; permit number 50/2011-B and number 612/2015-PR).

### 2.5. Dietary Supplementation and Testing Procedure

Mice were assigned to the following groups for each condition (young male, old male, young female, and old female): water (w), ethanol and glycerol (vehi), and Tilia (TTBEs). All the treatments were performed orally; vehi and TTBEs were dissolved in the drinking water (500 µL in 500 mL). We decided the route and timing of treatments to limit the stress of animals according to the nutraceutical paradigms. Animals were checked daily for the drugs intake and the weight was controlled before (day 0) and at the end (day 21) of the chronic administration.

### 2.6. Hole Board

The hole-board apparatus consists of black panel (40 × 40 cm, 2.2 cm thick) with 16 equidistant holes 3 cm in diameter in the floor. The board was positioned 15 cm above the table and divided into 9 squares of 10 × 10 cm. Each animal was placed singly in the center of the board and its behavior recorded with a video camera for 5 min. Head dippings, % of area explored, and the entries into the center were recorded according to [29]. Results are reported as means ± SEM.

### 2.7. Light/Dark Box

The light-dark box consists of two communicating sections one illuminated and the other dark (each comprising 35 × 30 × 21 cm). Each mouse was placed in the center of the light zone, and then the operator started to record 5 min of spontaneous exploration. Video recordings of mice behaviors were analyzed through the Tox Track software v2.83 [30].

### 2.8. Release Studies

Mice were sacrificed by cervical dislocation and promptly decapitated to collect the cortices. Each cortex was homogenized in 10 volumes of 0.32 M sucrose, buffered to pH 7.4 with Tris-(hydroxymethyl)-amino methane (Tris, final concentration (f.c.) 0.01 M) to prepare purified synaptosomes [31]. The homogenate was centrifuged at 1000× *g* for 5 min and the supernatant was stratified on a discontinuous Percoll gradient (2%, 6%, 10%, and 20% *v*/*v* in Tris-buffered sucrose) and centrifuged at 33,500× *g* for 5 min. The layer between 10% and 20% Percoll (synaptosomal fraction) was collected and washed by centrifugation. The synaptosomal pellets were resuspended in a physiological solution with the following composition (mM): NaCl, 140; KCl, 3; MgSO_4_, 1.2; CaCl_2_, 1.2; NaH_2_PO_4_, 1.2; NaHCO_3_, 5; HEPES, 10; glucose, 10; pH 7.2–7.4.

### 2.9. Experiments of Release

Purified nerve endings were incubated for 15 min a 37 °C with [3H]noradrenaline ([3H]NA; f.c.: 30 nM) in the presence of 0.1 µM 6-nitroquipazine and 0.1 µM GBR12909 to block false labelling of serotonergic and dopaminergic synaptosomes, respectively. An equal amount of synaptosomal suspension was then stratified on microporous filters at the bottom of parallel chambers in a Superfusion System (Ugo Basile, Comerio, Varese, Italy) [32] and maintained at 37 °C. Isolated nerve endings were superfused with the physiological solution for 38 min to balance the system and then exposed to muscimol. Eight minutes before agonist the synaptosomes were exposed to TTBEs or antagonists. Fractions and filters were collected as above and measured for radioactivity according to [33].

### 2.10. Statistical Analysis

The univariate statistical analysis and the correspondent graphical representation were carried out by using Past 3 or 4 [34]. An analysis of variance was performed by ANOVA followed by Tukey’s multiple comparison test. Data are presented as the mean ± standard error of the mean (SEM) and considered significant for *p* < 0.05 at least. Multivariate data analysis has been performed by CAT (Chemometric Agile Tool) software (August 27, 2020), one advanced chemometric multivariate analysis tool based on R, developed by the Chemistry Group of the Italian Chemical Society (http://gruppochemiometria.it/index.php/software). PCA was applied as explorative multivariate statistical method of unsupervised pattern recognition to rationalize the useful information of a data set [35,36]. Each column (variable) of the data set (data matrix) under study is considered as an axis in the multi-dimensional space and each row (object) of the data set under study is a point in this space. PCA algorithm searches for the maximum variance direction, the first principal component (PC1), corresponding to a high amount of information in the multidimensional space of the original data. The second principal component (PC2) is the direction having the largest remaining variance among all directions orthogonal to the first PC. As well as each succeeding component has as much of the remaining variability as possible which is not explained by the previous PCs. In this way, the large amounts of complex information, the multivariate original data matrix, is rationalized by way of simple bidimensional or tridimensional plots. Few animals were not included in the data when they showed lesions or complete immobility (*). Moreover, some (<3%) of video examinations were not included in the data analysis due to the video quality or software limits.

### 2.11. Drugs

1-[7,8 3H]-noradrenaline (specific activity 39 Ci mmol^−1^) was from Perkin Elmer. TTBEs from Gealpharma and muscimol bicucullin and all standards for HPLC analysis were from Sigma Aldrich (Milan, Italy). 6-Nitroquipazine maleate was donated from Duphar, Amsterdam, The Netherlands. 1-(2-(Bis-(4-fluorophenyl) methoxy) ethyl)-4-(3-phenylpropyl) piperazine dihydrochloride (GBR12909) was purchased from Tocris Bioscience (Bristol, UK). Ethanol was supplied by VWR International S.r.l (Milan, Italy).

## 3. Results

### 3.1. Bud-Extracts Characterization: UV-Visible and HPLC Fingerprints 

The quality control of the commercial bud-derivatives has been performed using both an untargeted spectroscopic fingerprint and a targeted chromatographic fingerprint as previously reported by the authors [37,38,39,40,41]. As far as the first one is concerned, UV-Visible spectroscopy was employed in a screening step in order to obtain a rapid preliminary untargeted fingerprint of the extract (after Standard Normal Variate—SNV pre-treatment) and followed by the targeted fingerprint by HPLC chromatography. Phytochemical fingerprint showed that benzoic acids (560.61 ± 31.92 mg/100 g FW) and catechins (424.20 ± 41.41 mg/100 g FW) represented the main phenolic classes in the analysed bud-extracts (47% and 36% of the total phytocomplex, respectively), followed by flavonols (166.34 ± 14.02 mg/100 g FW, 14% of the total phytocomplex) and cinnamic acids (32.02 ± 2.80 mg/100 g FW, 3% of the total phytocomplex) as reported in Table 1.

**Figure 1:**
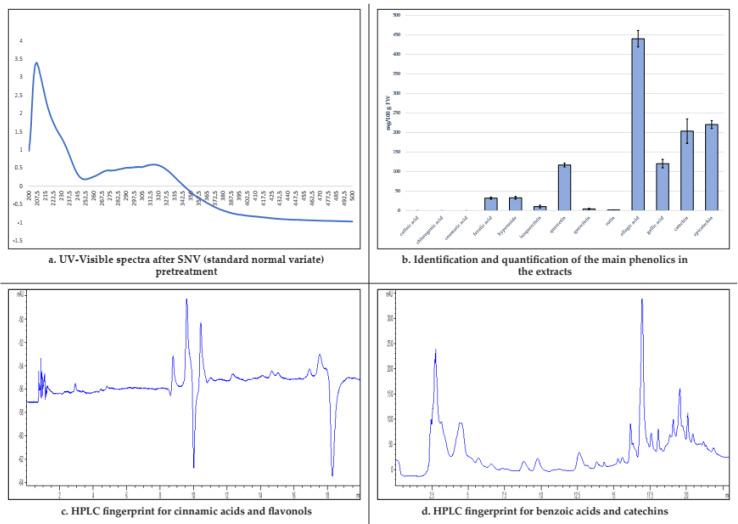
UV-Vis spectra (**a**), bioactive compound quantification (**b**), and HPLC fingerprint (**c**,**d**) of the *T. tomentosa* bud-extracts.

In Figure 1, UV-Visible spectra, HPLC fingerprint, and bioactive compound quantification of the *T. tomentosa* bud-extract were reported.

### 3.2. Evaluation Active Dilutions of TTBEs on Native Brain Targets

**Figure 2:**
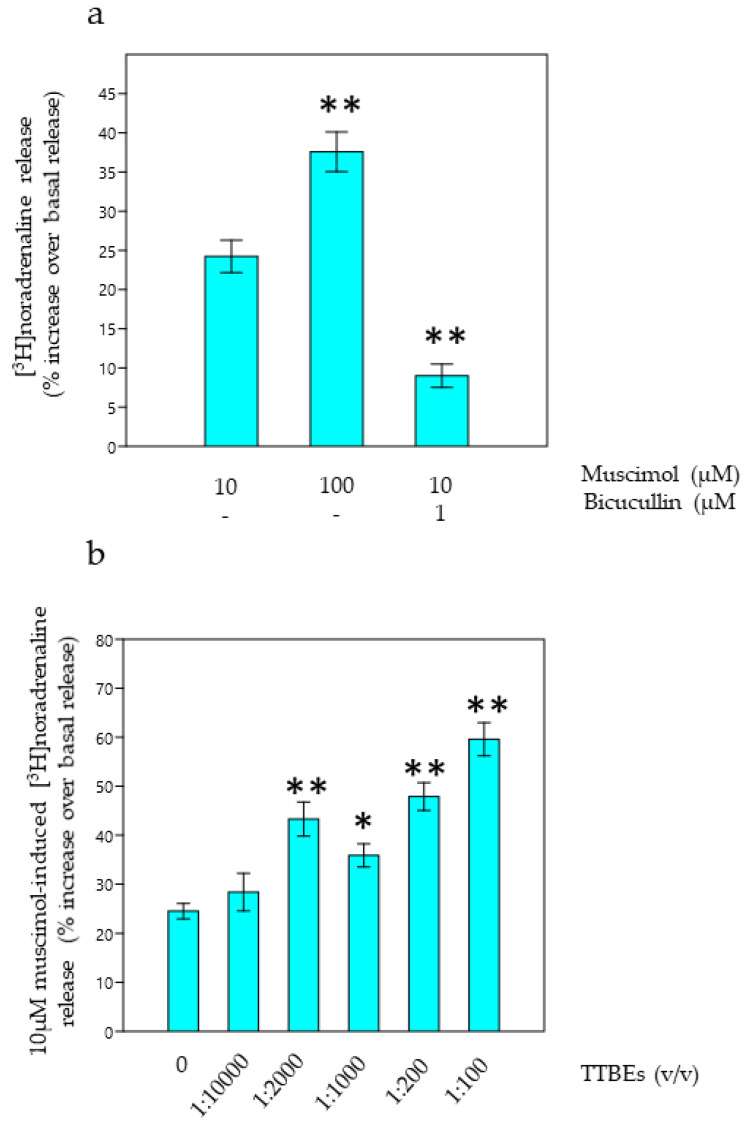
In vitro noradrenaline release from cortical synaptosomes of young male mice: functional activity of native GABAA receptors in presence of TTBEs dilutions. (**a**) Counteracting effect of 1 µM bicucullin on muscimol evoked [3H]noradrenaline release (**b**). Effects of TTBEs on 10 µM muscimol evoked [3H]noradrenaline release. Data represent the mean ± S.E.M. from six young male mice. Statistical analysis was performed by applying ANOVA followed by the Tukey’s Multiple Comparison test. * *p* < 0.05, ** *p* < 0.01 vs. 10 µM muscimol.

Cortical noradrenergic synaptosomes express functional GABAA receptors modulating noradrenaline exocytosis as previously demonstrated in rats by Schmid and colleagues in 1999. Accordingly, in our experimental conditions, muscimol induces a concentration-dependent stimulation of these receptors able, in cascade, to elicit noradrenaline release in a bicucullin-sensitive manner (1 µM; Figure 2a). Data in the literature, published adopting an electrophysiological approach, suggest that TTBEs mimic GABA and benzodiazepines at GABAA receptors [20]. Consequently, we selected a submaximal concentration of muscimol to investigate the modulatory role of TTBEs on presynaptic GABAA receptors regulating noradrenaline release. Progressive water-dilutions of commercial bud extracts are utilized during in vitro superfusion experiments from cortical synaptosomes. Figure 2b demonstrates that TTBEs potentiated the 10 µM muscimol induced noradrenaline release in a concentration-dependent manner. Interestingly, 1:2000 TTBEs provoked a significant increase in the muscimol induced [3H] noradrenaline release that is also confirmed at lower dilutions. Interestingly, 1:2000 TTBEs was ineffective on noradrenaline basal release (data not shown). 

### 3.3. Dietary Supplementation with TTBEs: Control of Daily Intake and Weight Gain 

Basing on in vitro results, we start with the in vivo behavioural evaluations of anxiolytic TTBEs properties. A total of 21 days of chronic oral administration of 1:2000 TTBEs in drinking water involved 4 animal groups divided according to gender and age profile (young male, young female, old male, and old female). Moreover, each group is separated into three subgroups based on the drinking solutions (water, vehicle, and TTBEs) to accurately discriminate possible effects due to the presence of ethanol and glycerin. During the oral supplementation, animals are monitored to evaluate changes in water consumption and body weight. Data displayed in Table 2 demonstrated that neither the vehicle nor the TTBEs significantly modify the amount of liquid drunk. Accordingly, in each group, the weight growth was significantly unaltered by vehicle or TTBEs.

### 3.4. Dietary Supplementation with TTBEs: Behavioral Scores 

Animal behavioral performances in the hole board test (n° head dippings—%HD, % of area explored—%AE, % of entries into center—%C) and in light dark box test (time in light—tL, n° of transitions—T, average speed—Av Sp, exploration rate %—RAE and total distance—D) are analyzed before (day 0) and at the end (day 21) of the dietary supplementation.

The corresponding data matrix D_16,8_ has been reported in Table 3.

In details, the data matrix D_16,8_, whose rows are the objects (the first 12 correspond to the treated animals and the last 4 to the animals at the zero conditions) and whose columns are the results of the 8 behavioral test investigated, was taken into account. The objects are described by 8 experimental variables and by 2 categories (c1: age and c2: sex). PCA was applied as unsupervised pattern recognition technique in order to explore the mice behavior information using a multivariate approach [35,36]. PCA was performed after the autoscaling pre-processing of the data matrix in order to elaborate the multivariate data characterized by different scales and units. This pre-processing technique commonly used in multivariate analysis, consists of mean-centering followed by the division of each column of the data matrix by its standard deviation [35]. 

**Figure 3:**
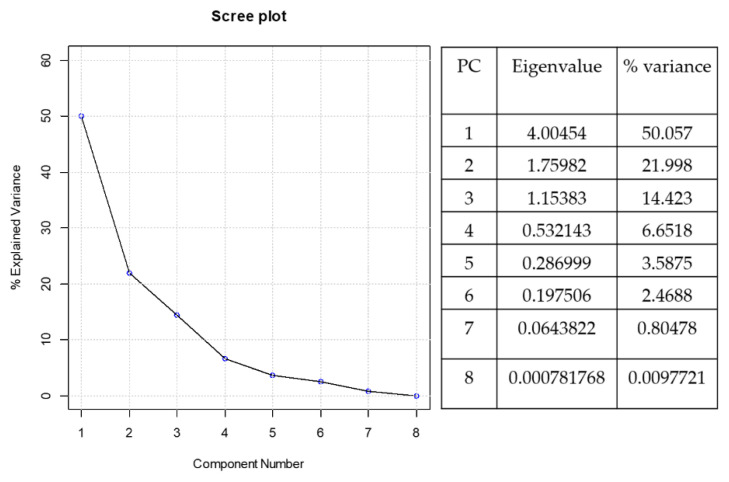
Scree plot: % explained variance of each PCs.

The first 2 principal components (PC1-PC2) explained the 72% of the total variance/information of the data set as highlighted in Figure 3. 

**Figure 4:**
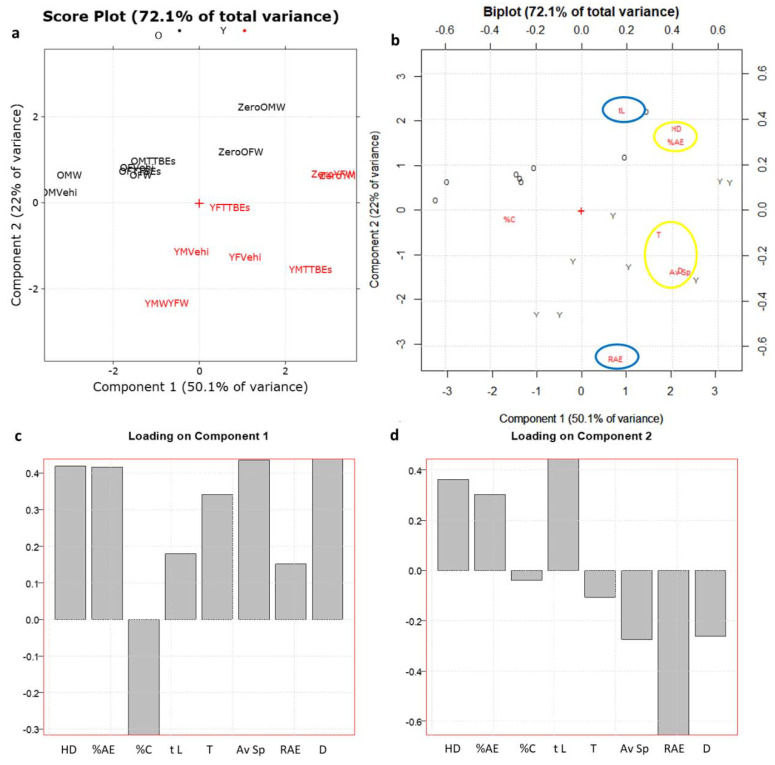
(**a**) PC1-PC2 score plot and biplot of the D_16,8_ data matrix. (**b**) PC1-PC2 biplot (scores plus loadings plot). Animals are categorized by age: old mice are reported in black and young ones in red, respectively. (**c**) Loading plot on PC1. (**d**) Loading plot on PC2. YMW: young male water, YFW: young female water, YMVehi: young male vehicle, YFVehi: young female vehicle, YMTTBEs: young male *Tilia tomentosa* Moench Buds Extracts, YFTTBEs: young female *Tilia tomentosa* Moench Buds Extracts, ZeroYMW: day 0 young male water, ZeroYFW: day 0 young female water, OMW: old male water, OFW: old female water, OMVehi: old male vehicle, OFVehi: old female vehicle, OMTTBEs: old male *Tilia tomentosa* Moench Buds Extracts, OFTTBEs: old female *Tilia tomentosa* Moench Buds Extracts, ZeroOMW: day 0 old male water, ZeroOFW: day 0 old female water, HD: n° head dippings, %AE: % of area explored, %C: % of entries into center, tL: time in light, T: n° of transitions, Av Sp: average speed, RAE: exploration rate %, D: total distance.

Figure 4 shows the PCA score plot, biplot (scores plus loadings plot), and loading plots on the first to the second PCs obtained from the above-mentioned data matrix. 

PC1, the direction of maximum variance which explains the 50.1% of the total information, allows a good discrimination between the objects according to the different treatments (W: water; Vehi: vehicle; TTBES: Tilia). Particularly, as shown in Figure 4a, TTBEs move objects to the animals at the starting conditions (indicated as zero) which have higher scores on PC1. Regarding young animals (in red), these zero conditions are the same both for male and for female, while in the case of old mice (in black) are different between the genders. 

**Figure 5:**
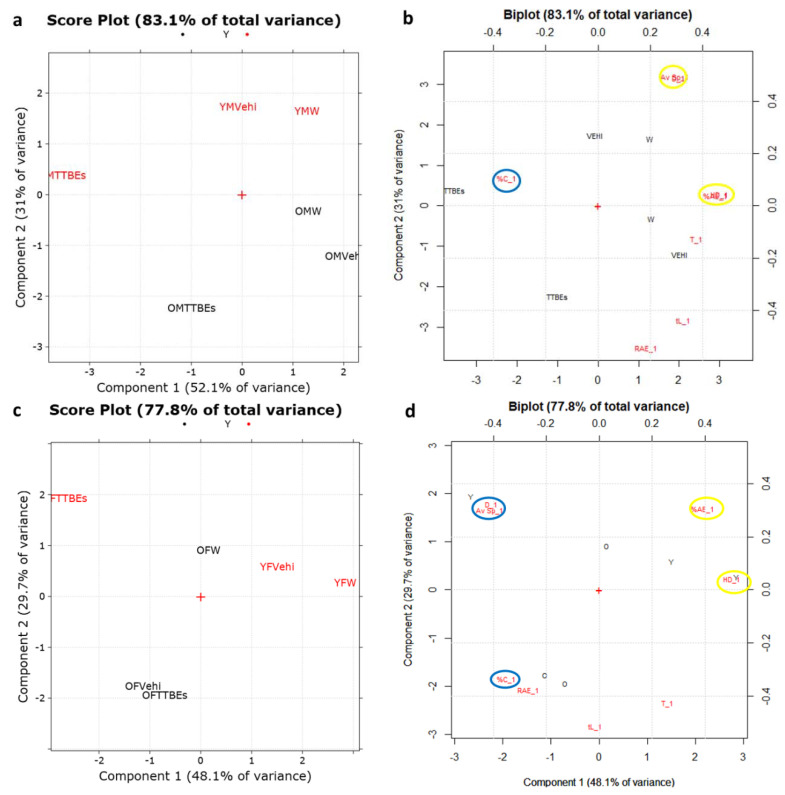
PC1-PC2 score plot (**a**) and biplot (**b**) of the M_16,8_ data matrix. PC1-PC2 score plot (**c**) and biplot (**d**) of the F_16,8_ data matrix. tL_1: time in light day 21-day 0, HD_1: head dippings day 21-day 0, AE_1: % of area explored day 21-day 0, RAE_1: exploration rate % day 21-day 0, %C_1: % of entries into center day 21-day 0, Av Sp_1: average speed day21-day0, T_1: n° of transitions day 21-day 0, D_1: distance day 21-day 0, W: water, Vehi: vehicle, TTBEs: *Tilia tomentosa* Moench Buds Extracts.

On PC2, which explains the 22% of the remaining variance, the old animals (in black) are separated from the young ones (in red). As showed in Figure 4b,c, the HD (n° of head dippings), the %AE (% of area explored), D (total distance) and Av Sp (average speed), highlighted in yellow in the biplot, had highest loadings on PC1. Instead, variables obtained from the light dark box test such as the time in light (tL) and the exploration rate (RAE), highlighted in blue in the biplot, had highest loadings on PC2 and they resulted important to separate young and old animals (Figure 4d). The PCA analysis of our data clearly demonstrated that young and old animals are very discernible by the variable included. Indeed, the age-dependent behaviors are strongly described by the PC2 that is positively associated with tL and RAE. According to previous results, repeated exposure to a novel apparatus produced a reduction in exploration parameters due to a process commonly referred to as habituation [42]. Indeed, in our experimental conditions, most of the behavioral parameters were decreased in water groups compared with the scores in time 0 section (Table 3). Curiously, the habituation seemed more effective in the young male group and less potent in the old female group. Across the age and gender groups, this phenomenon appeared less active on two parameters (%C and RAE). To precisely evaluate the effects of the oral supplementation and the gender dependency, we decided to normalize each animal score, subtracting the respective time 0 section scores. In detail, two separate matrices were created (M_6,8_ for male mice and F_6,8_ for female ones, respectively) by subtracting the corresponding zero condition for each object (Figure 5).

Figure 5a shows the PC1-PC2 score plot of M_6,8_ data matrix corresponding to the male animals, which explains the 83.1% of the total variance/information of the data set. In this plot, the effects of TTBEs treatment on young (in red) and old (in black) mice, respectively, are better highlighted. In fact, both for young and old mice, the TTBEs animals separate on PC1 from the corresponding ones treated with W or Vehi. On PC2, as previously already described, old animals (in black, higher scores on PC2) are separated from the young ones (in red, lower scores on PC2). After the TTBEs treatment the HD, % AE, Av Sp, and D variables increase (Figure 5b) and consequently the differences with respect to the zero conditions decrease, i.e., HD_1, % AE_1, Av Sp_1, and D_1 (highlighted in yellow). On the contrary as concerns %C variable, the difference with respect to the zero conditions (i.e., %C_1 highlighted in blue) increases.

As regards young females (in red), the PC1-PC2 score plot of F_6,8_, which almost explains the 78% of the total variance, shows a similar trend to male mice (Figure 5c). Particularly after the TTBEs treatment, the HD and % AE increase (Figure 5d, loadings highlighted in yellow) while % C, Av Sp, and D decrease determining an increase in differences compared to the corresponding zero condition (i.e., % C_1, Av Sp_1, D_1: in blue). The two obtained distinct matrices for male and female animals are reported in Appendix A. 

**Figure 6:**
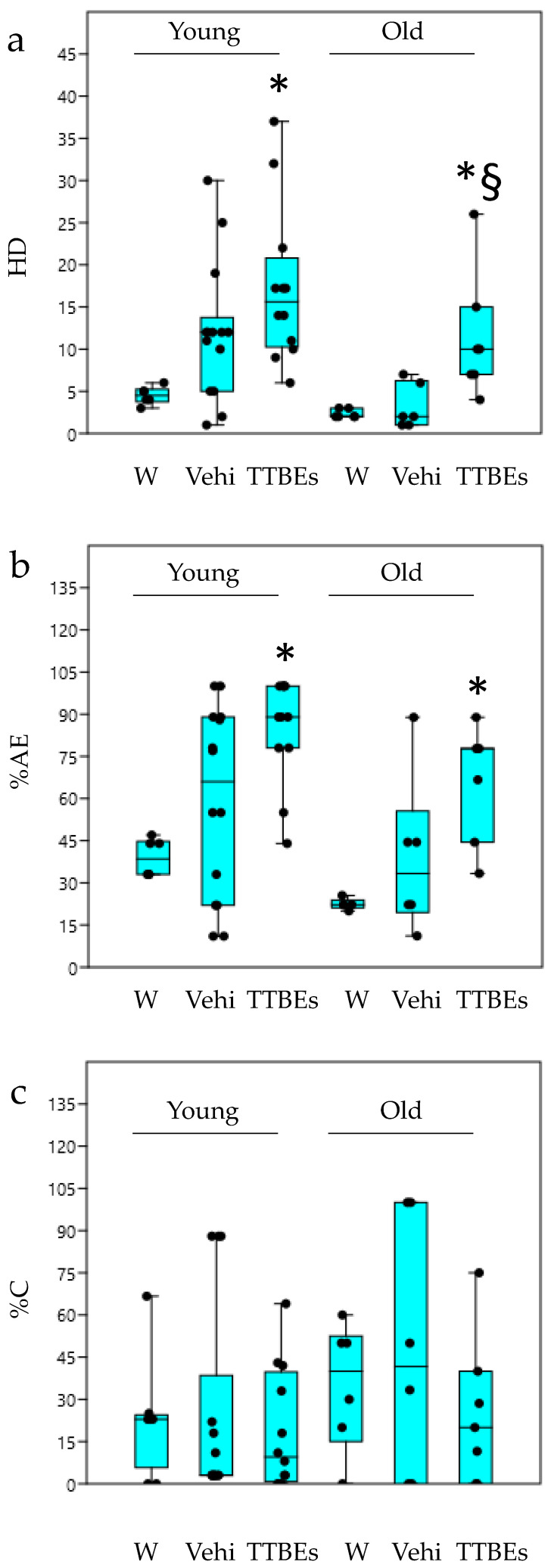
Hole Board test in young and old male mice: effects 21 days of dietary integration with vehicle, TTBEs respect to water. Behavioral skills were quantified as (**a**) n° head dippings (%HD), (**b**) % of area explored (%AE), (**c**) % of entries into center (%C). Data represent the mean ± S.E.M. (*n* = 6 (W), 14 (Vehi) 12 (TTTBEs) young and (*n* = 6 (W), 6 (Vehi) 6 (TTTBEs) old mice analyzed in three different trials. Statistical analysis was performed by applying ANOVA followed by the Tukey’s multiple comparison test. * *p* < 0.05; *p* < 0.01 vs. water; ^§^ *p* < 0.05 vs. vehicle.

**Figure 7:**
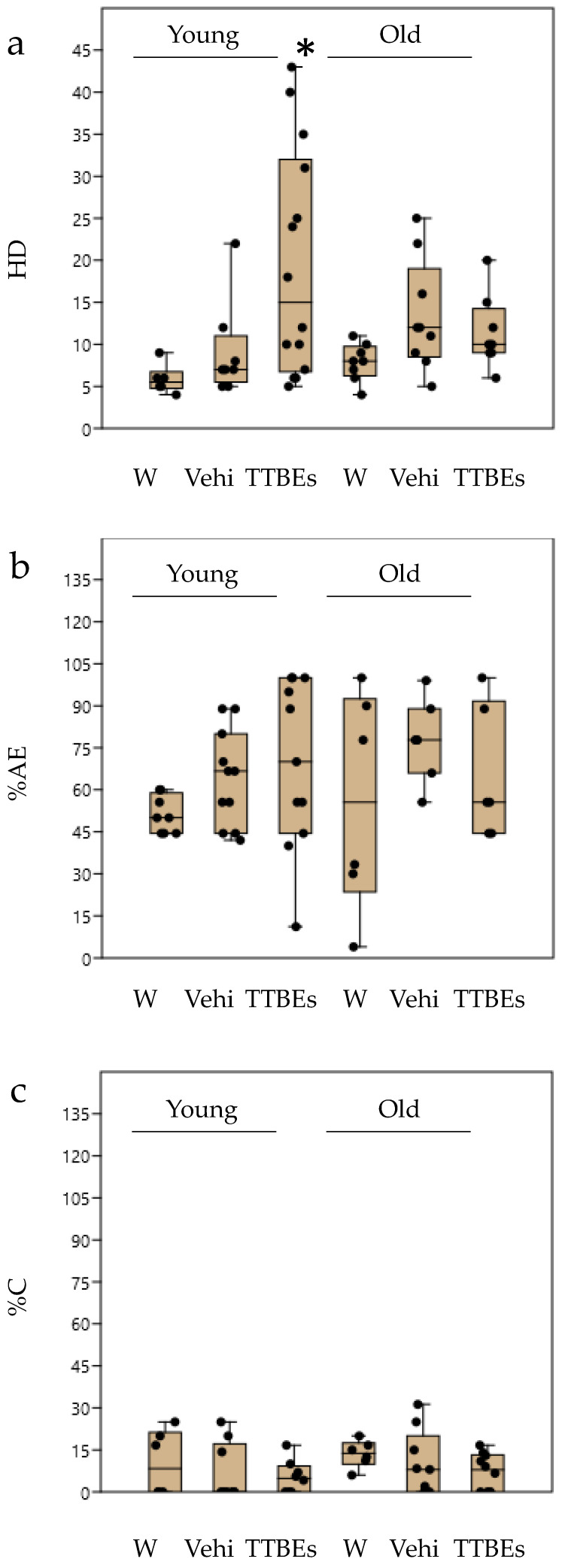
Hole Board test in young and old female mice: effects 21 days of dietary integration with vehicle, TTBEs respect to water. Behavioral skills were quantified as (**a**) n° head dippings (%HD), (**b**) % of area explored (%AE), (**c**) % of entries into center (%C). Data represent the mean ± S.E.M. (*n* = 6 (W), 11 (Vehi) 14 (TTTBEs) young and (*n* = 8 (W), 9 (Vehi) 9 (TTTBEs) old mice analyzed in three different trials. Statistical analysis was performed by applying ANOVA followed by the Tukey’s multiple comparison test. * *p* < 0.05 water.

Then we performed a post-hoc analysis on each behavioural variable to better characterize the anxiolytic effect of TTBEs. It has been previously reported that the exposure of animals to various stressful stimuli decreases some exploratory behaviours [43,44]. In the hole-board test, a pronounced inhibition of head-dipping behaviour was observed in animals that had been exposed to stressful stimuli [45]. The results with the univariate statistical analysis demonstrated a significant increase in the number of head dippings in young animals and old male mice after 21 days of TTBEs treatment (Figure 6a). Moreover, the chronic oral treatment with bud extracts increased the total amount of locomotion, expressed as % of area explored (Figure 6b), both in young and in old male mice. Although the TTBEs did not increase the %center entries (Figure 6c), both curiosity and exploration were modified which is consistent with a reduction in anxiety-like behaviour. Conversely, the HB scores in females are insensitive to dietary integration with the exception of HD in younger animals (Figure 7a).

**Figure 8:**
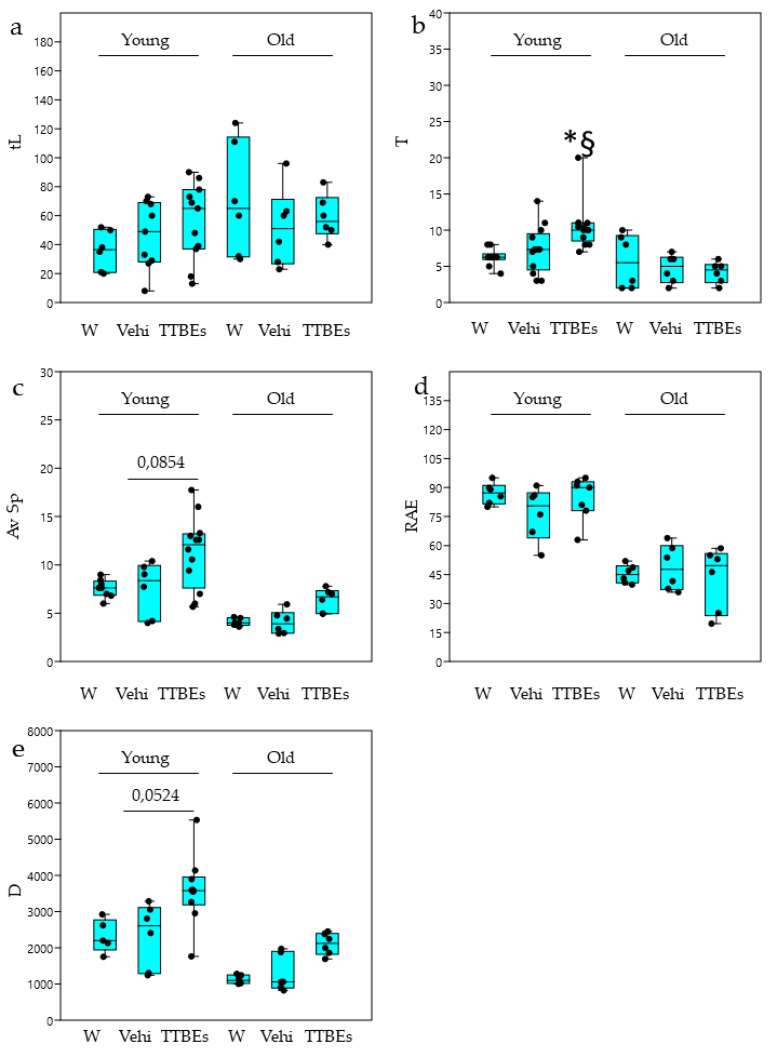
Light Dark test in young and old male mice: effects 21 days of dietary integration with vehicle, TTBEs respect to water. Behavioral skills were quantified as (**a**) time in light (tL), (**b**) n° of transitions (T), (**c**) average speed (Av Sp), (**d**) exploration rate % (RAE) and (**e**) total distance (D). Data represent the mean ± S.E.M. (*n* = 9 (W), 12(Vehi) 12 (TTTBEs) young and (*n* = 6 (W), 6 (Vehi) 6 (TTTBEs) old mice analyzed in three different trials. Statistical analysis was performed by applying ANOVA followed by the Tukey’s multiple comparison test. * *p* < 0.05 water; ^§^ *p* < 0.05 vs. vehicle.

**Figure 9:**
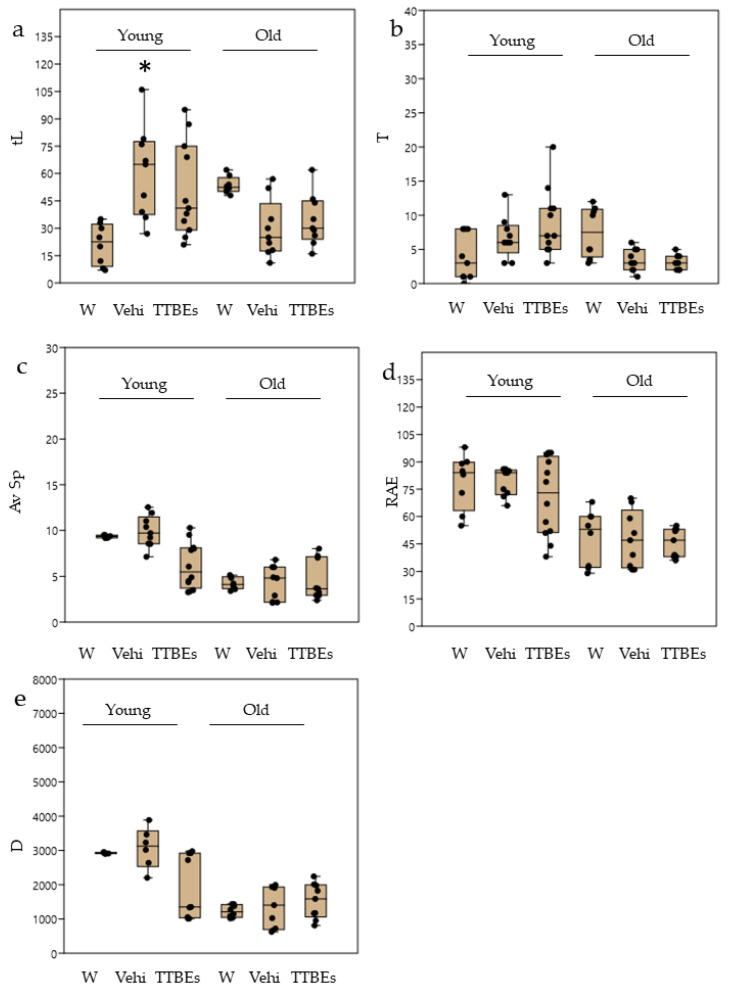
Light Dark test in young and old female mice: effects 21 days of dietary integration with vehicle, TTBEs respect to water. Behavioral skills were quantified as (**a**) time in light (tL), (**b**) n° of transitions (T), (**c**) average speed (Av Sp), (**d**) exploration rate % (RAE) and (**e**) total distance (D). Data represent the mean ± S.E.M. (*n* = 8 (W), 9 (Vehi) 12 (TTTBEs) young and (*n* = 6 (W), 9 (Vehi) 9 (TTTBEs) old mice analyzed in three different trials. Statistical analysis was performed by applying ANOVA followed by the Tukey’s multiple comparison test. * *p* < 0.05 water.

In the LD paradigm, TTBEs dietary supplementation produced a significant increase in transitions in young male mice coupled with a positive trend in the speed and distance scores (Figure 8). Conversely, the oral administration of Tilia extracts did not significantly modify the LD score in female mice (Figure 9). Interestingly, some of the LD parameters appeared modified by the consumption of vehicle, confirming a susceptibility of females to ethanol.

## 4. Discussion

Accumulating evidence from animal and human research reinforce the concept of the microbiome–gut–brain axis. Indeed, microbiome regulates acknowledged functions of the CNS, the immunity system and behavior in health and disease. Moreover, the diet may modulate gut microbiome, altering the nutrient availability [46]. Interestingly, anxiety disorders are common and complex psychiatric syndromes susceptible to the influence of microbiome signaling [47,48]. This aspect is extremely important considering the global number of people affected by stress-related disorders. Curiously, some categories seem to be more susceptible as documented by the literature showing that aging typically produces an increase in anxiety vulnerability and sedative consumption [49,50,51,52]. Furthermore, gender vulnerability seems to emerge in a context of great complexity [53,54]. If this is not enough, the approved pharmacological treatments cause severe side effects like hallucinations, addiction, suicide, insomnia, and loss of motor coordination. Therefore, alternative strategies that combine different approaches are fundamental in line to resolve some of these peculiarities. Dietary integration with nutraceutical supplements could represent an intriguing plan for improving the efficacy and the safety of synthetic anxiolytics. At this regard, we decided to investigate an in vivo oral supplementary protocol with commercial buds extracts of *Tilia Tomentosa* in rodents combining the analysis of different behavioral parameters. In details, we studied the role of aging, gender, and nutrients, alone or in combination, on the stress-related response of C57BJ mice. Our results demonstrated that 21 days of dietary integration with TTBEs produce anxiolytic effects in mice. This evidence is in accordance with previous in vitro results describing GABA and benzodiazepine-like actions evoked by TTBEs [20]. Moreover, our research confirms the anxiolytic properties of active compounds present in the biological matrix of *Tilia Tomentosa* [19,55,56,57,58,59]. To be specific, results presented here demonstrate that active nutrients, extracted from buds and freely administered through the drunk water, can modify mice behavior with gender and age specificity. Interestingly, our monitoring of liquid consumption per day revealed that dietary integration with TTBEs are well accepted and all the animals do not change the hydration rate. Accordingly, the different mice groups displayed a weight trend in line with the respective water groups. Combined statistical analysis revealed that male mice seemed more sensitive to the natural supplements modifying both motor and curiosity score. Interestingly, young mice demonstrated the maximum performance increase both in HB and LD test. Old male mice reached a significant increase in the number of HD and the %AE but fail to ameliorate the LD test parameters. These results need further investigations focused, among other things, on the diverse composition of the gut microbiome [60,61,62] and the diverse sensitivity for light in ancient mice [63]. On the other hand, we cannot exclude the possibility that increasing concentrations of TTBEs or a longer time of supplementation can be effective. However, in line with the nutraceutical standpoint, we decided to administrate TTBEs at the maximum dilution rate according to our in vitro results on native brain targets. Accordingly, we do not have performed the gavage to avoid additional stress stimuli. Interestingly, the analysis of female trends reveals a different sensitivity to TTBEs. Old females seem to be completely unaffected by dietary supplementation. This evidence reinforces the idea, based on male results, that aging modifies animal sensitivity to TTBEs. Likewise, young female reveals low sensitivity to supplements, showing only a positive trend in the HB results. We are aware that results from rodents may not be equivalent to those in human. Although, TTBEs are commercial products commonly integrated in the human diet according to the paradigms of traditional medicine. The generalized lack of efficacy in female mice could be explained in part to the action of vehicle constituents. Indeed, water and vehicle groups are fundamental controls fixed in our protocol to elucidate the impact of glycerol and ethanol on mice. Ethanol is a potent modulator of GABAA receptors, and some studies have convincingly demonstrated the sedative effects both in male and female rodents [63,64,65]. The LD score of female groups describes a predictable modulation of behaviors mediated by vehicle. We observed a common trend for TTBEs and vehicle in two LD parameters (tL and T) and a different trend in (Av Sp and D). Interestingly, PCA analysis also indicated that vehicle closely resembles TTBEs in old females. Although not significative, these data support the idea that low doses of ethanol could modify the LD scores in females. Indeed, females possess enzymatic and hormonal characteristics enhancing ethanol influence [66,67,68]. Furthermore, estrogens also interfere with anxiety and motor performances [69,70,71]. Future research will be dedicated to correlate the female response to their hormonal profile.

On the other hand, we cannot exclude the possibility that bioactive compounds present in TTBEs produce diverse actions on the basis of the animal peculiarities. Furthermore, we must keep in mind that TTBEs are a mixture of different active compounds extracted from a vegetal matrix. The efficacy of these supplements is strictly linked to the presence of standard quality control. In this regard, we also describe a bioactive compounds quantification of TTBEs nutrients revealing a remarkable presence of catechins, benzoic acids, and flavonols. Interestingly, many of the substances detected, like quercetin, ellagic acid, gallic acid, and catechin, are also associated with anxiolytic effects in the literature [72,73,74,75,76,77,78] but a detailed assessment of the bioavailability of these compounds by gender and age in mice is still lacking.

## 5. Conclusions

In summary, dietary integration with TTBEs reduce anxiety-related behavior in mice showing different efficacy depending on the age and gender characteristics and without apparent side effects. Moreover, our data highlight the importance of limiting ethanol concentration waiting to determinate the precise mechanism of action. Overall taking into account the results, we described for the first time the efficacy of TTBEs after oral consumption. Finally, our results support the idea that an integration of human diet with botanicals could represent an improvement in the therapeutic protocols of anxiety disorders.

## Figures and Tables

**Table 1:** Main phytochemicals in *T. tomentosa* bud-extract.

Chemical Class	Phytochemical	mg/100 g of Fresh Weight (FW)
Cinnamic acids	Caffeic acid	n.d.
	Chlorogenic acid	n.d.
	Coumaric acid	n.d.
	Ferulic acid	32.02 ± 2.80
Flavonols	Hyperoside	32.98 ± 3.46
	Isoquercitrin	10.45 ± 3.67
	Quercetin	116.75 ± 4.95
	Quercitrin	4.38 ± 1.59
	Rutin	1.78 ± 0.36
Benzoic acids	Ellagic acid	440.21 ± 21.02
	Gallic acid	120.40 ± 10.90
Catechins	(+)-Catechin	203.56 ± 31.39
	(−)-Epicatechin	220.64 ± 10.03

The results are expressed as mean ± S.D. n.d. = not detected.

**Table 2:** Weight gain and water consumption.

Animals	Treatment	Water Consumption	Weight			*n*
			Start	End	Variation	
YM	W	6.38 ± 0.63	26.4 ± 0.3	27.1 ± 0.3	(+2.8%)	15
	Vehi	6.12 ± 0.55	27.9 ± 0.5	29 ± 0.6	(+4.3%)	26
	TTBEs	6.91 ± 0.26	27 ± 0.2	28.3 ± 0.3	(+5%)	24
YF	W	5.94 ± 0.45	22 ± 0.2	22.5 ± 0.3	(+2.3%)	14
	Vehi	5.67 ± 0.38	21.7 ± 0.2	22.6 ± 0.1	(+4.1%)	20
	TTBEs	5.39 ± 0.58	20.5 ± 0.1	20.9 ± 0.1	(+2.2%)	26
OM	W	6.65 ± 0.13	29.2 ± 0.5	29.2 ± 0.4	(−0.1%)	12
	Vehi	6.12 ± 0.48	30.6 ± 0.5	30.5 ± 0.5	(−0.2%)	12
	TTBEs	6.78 ± 0.19	33.6 ± 0.4	33.7 ± 0.3	(+0.3%)	12
OF	W	4.91 ± 0.62	25.6 ± 0.5	25.8 ± 0.5	(+0.8%)	14
	Vehi	4.37 ± 0.52	26.5 ± 0.3	26.2 ± 0.2	(−1.4%)	18
	TTBEs	3.83 ± 0.33	26.7 ± 0.3	26.3 ± 0.4	(−1.7%)	18

The results are expressed as mean ± S.E.M. YM: young male, YF: young female, OM: old male, OF: old female, W: water, Vehi: vehicle, TTBEs: *Tilia tomentosa* Moench Buds Extracts.

**Table 3:** Data matrix D_16,8_: 16 animal groups, classified both by age (c1) and gender (c2), and the 8 behavioral scores.

	c1	c2	%HD	%AE	%C	tL	T	Av Sp	RAE	D
YMW	Y	M	4.50	38.89	22.92	36.00	6.25	7.62	87.60	2375.00
YMVehi	Y	M	12.00	59.26	24.37	46.00	7.33	7.52	76.67	2350.00
YMTTBEs	Y	M	17.22	83.95	18.88	56.00	10.45	11.55	84.42	3586.00
YFW	Y	F	5.83	50.00	10.28	21.25	3.75	9.34	79.50	2918.00
YFVehi	Y	F	9.12	63.89	6.59	60.11	6.78	9.88	78.83	3074.00
YFTTBEs	Y	F	19.75	69.44	5.42	50.82	9.00	6.15	70.60	1920.00
OMW	O	M	2.33	22.22	33.33	71.00	5.50	4.10	45.20	1124.00
OMVehi	O	M	3.17	38.89	47.22	51.50	4.67	4.05	48.67	1282.00
OMTTBEs	O	M	11.29	66.67	25.01	59.00	4.25	6.38	43.00	2104.00
OFW	O	F	7.67	55.00	13.42	52.50	7.67	4.21	48.00	1224.00
OFVehi	O	F	13.80	75.55	9.51	30.00	3.33	4.31	47.00	1358.00
OFTTBEs	O	F	11.17	70.37	7.26	35.16	3.60	4.70	46.00	1527.00
ZeroYMW	Y	M	30.41	94.95	5.73	58.69	8.23	12.54	46.75	3911.75
ZeroYFW	Y	F	31.42	93.98	8.15	71.38	8.00	11.24	60.50	3508.50
ZeroOMW	O	M	28.35	93.46	12.63	95.67	6.22	6.97	55.25	2210.00
ZeroOFW	O	F	27.00	82.64	13.87	58.31	7.07	7.19	50.67	2309.17

YMW: young male water, YFW: young female water, YMVehi: young male vehicle, YFVehi: young female vehicle, YMTTBEs: young male *Tilia tomentosa* Moench Buds Extracts, YFTTBEs: young female *Tilia tomentosa* Moench Buds Extracts, ZeroYMW: day 0 young male water, ZeroYFW: day 0 young female water, OMW: old male water, OFW: old female water, OMVehi: old male vehicle, OFVehi: old female vehicle, OMTTBEs: old male *Tilia tomentosa* Moench Buds Extracts, OFTTBEs: old female *Tilia tomentosa* Moench Buds Extracts, ZeroOMW: day 0 old male water, ZeroOFW: day 0 old female water.

## Appendix A

**Table A1:** Data matrix M_6,8_: male mice.

	c1	c2	HD_1	%AE_1	%C_1	tL_1	T_1	Av Sp_1	RAE_1	D_1
YMW	Y	M	25.90909	56.05949	−17.1947	22.69231	1.980769	4.915	−40.85	1536.75
YMVehi	Y	M	18.40909	35.68949	−18.6447	12.69231	0.900769	5.015	−29.92	1561.75
YMTTBEs	Y	M	13.18909	10.99949	−13.1547	2.692308	−2.21923	0.985	−37.67	325.75
OMW	O	M	26.02294	71.24405	−20.6994	24.66667	0.72	2.865	10.05	1086
OMVehi	O	M	25.18294	54.57405	−34.5894	44.16667	1.55	2.915	6.58	928
OMTTBEs	O	M	17.06294	26.79405	−12.3794	36.66667	1.97	0.585	12.25	106

**Table A2:** Data matrix F_6,8_: female mice.

	c1	c2	HD_1	%AE_1	%C_1	tL_1	T_1	Av Sp_1	RAE_1	D_1
YFW	Y	F	25.58667	43.98148	−2.12525	50.125	4.25	1.895	−19	590.5
YFVehi	Y	F	22.29667	30.09148	1.564749	11.265	1.22	1.355	−18.33	434.5
YFTTBEs	Y	F	11.66667	24.54148	2.734749	20.555	−1	5.085	−10.1	1588.5
OFW	O	F	20.68294	38.46405	−0.78936	43.16667	−1.45	2.755	7.25	986
OFVehi	O	F	14.55294	17.91405	3.120638	65.66667	2.89	2.655	8.25	852
OFTTBEs	O	F	17.18294	23.09405	5.370638	60.50667	2.62	2.265	9.25	683

## References

[1] Foster J.A., McVey Neufeld K.-A. (2013). Gut-brain axis: How the microbiome influences anxiety and depression. Trends Neurosci..

[2] Pandey M., Verma R.K., Saraf S.A. (2010). Nutraceuticals: New era of medicine and health. Asian J. Pharm. Clin. Res..

[3] Olatunji B.O., Wolitzky-Taylor K.B. (2009). Anxiety sensitivity and the anxiety disorders: A meta-analytic review and synthesis. Psychol. Bull..

[4] Lewinsohn P.M., Lewinsohn M., Gotlib I.H., Seeley J.R., Allen N.B. (1998). Gender differences in anxiety disorders and anxiety symptoms in adolescents. J. Abnorm. Psychol..

[5] Depaola S.J., Griffin M., Young J.R., Neimeyer R.A. (2003). Death anxiety and attitudes toward the elderly among older adults: The role of gender and ethnicity. Death Stud..

[6] Lynch S.M. (2000). Measurement and Prediction of Aging Anxiety. Res. Aging.

[7] Cazard F., Ferreri F. (2013). Troubles bipolaires et troubles anxieux comorbides: Impact pronostique et enjeux therapeutiques. L’encephale.

[8] Meisel M.K., Goodie A.S. (2015). Predicting prescription drug misuse in college students’ social networks. Addict. Behav..

[9] Curran S., Musa S., Sajjadi A. (2015). Hypnosedatives and Anxiolytics. Side Effects of Drugs Annual.

[10] Basile A.S., Lippa A.S., Skolnick P. (2004). Anxioselective anxiolytics: Can less be more?. Eur. J. Pharm..

[11] Farach F.J., Pruitt L.D., Jun J.J., Jerud A.B., Zoellner L.A., Roy-Byrne P.P. (2012). Pharmacological treatment of anxiety disorders: Current treatments and future directions. J. Anxiety Disord..

[12] Alramadhan E., Hanna M.S., Hanna M.S., Goldstein T.G., Avila S.M., Weeks B.S. (2012). Dietary and botanical anxiolytics. Med. Sci. Monit..

[13] Calixto J.B. (2000). Efficacy, safety, quality control, marketing and regulatory guidelines for herbal medicines (phytotherapeutic agents). Braz. J. Med. Biol. Res..

[14] Konik E.A., Jungling R.C., Bauer B.A. (2011). Herbs and Dietary Supplements in the European Union: A Review of the Regulations with Special Focus on Germany and Poland. J. Diet. Suppl..

[15] Dell’agli M., Di Lorenzo C., Badea M., Sangiovanni E., Dima L., Bosisio E., Restani P. (2013). Plant Food Supplements with Anti-Inflammatory Properties: A Systematic Review (I). Crit. Rev. Food Sci. Nutr..

[16] Gulati O.P., Berry Ottaway P., Coppens P. (2014). Botanical Nutraceuticals, (Food Supplements, Fortified and Functional Foods) in the European Union with Main Focus on Nutrition And Health Claims Regulation. Nutraceutical and Functional Food Regulations in the United States and Around the World.

[17] Ieri F., Innocenti M., Possieri L., Gallori S., Mulinacci N. (2015). Phenolic composition of “bud extracts” of *Ribes nigrum* L., *Rosa canina* L. and *Tilia tomentosa* M. J. Pharm. Biomed. Anal..

[18] Donno D., Beccaro G.L., Mellano M.G., Bonvegna L., Bounous G. (2014). Castanea spp. buds as a phytochemical source for herbal preparations: Botanical fingerprint for nutraceutical identification and functional food standardisation. J. Sci. Food Agric..

[19] Viola H., Wolfman C., de Stein M.L., Wasowski C., Peña C., Medina J.H., Paladini A.C. (1994). Isolation of pharmacologically active benzodiazepine receptor ligands from *Tilia tomentosa* (Tiliaceae). J. Ethnopharmacol..

[20] Allio A., Calorio C., Franchino C., Gavello D., Carbone E., Marcantoni A. (2015). Bud extracts from *Tilia tomentosa* Moench inhibit hippocampal neuronal firing through GABAA and benzodiazepine receptors activation. J. Ethnopharmacol..

[21] Mohler H. (2006). GABA A Receptors in Central Nervous System Disease: Anxiety, Epilepsy, and Insomnia. J. Recept. Signal Transduct..

[22] Kalueff A.V., Nutt D.J. (2007). Role of GABA in anxiety and depression. Depress. Anxiety.

[23] Goddard A.W., Ball S.G., Martinez J., Robinson M.J., Yang C.R., Russell J.M., Shekhar A. (2010). Current perspectives of the roles of the central norepinephrine system in anxiety and depression. Depress. Anxiety.

[24] Nuss P. (2015). Anxiety disorders and GABA neurotransmission: A disturbance of modulation. Neuropsychiatr. Dis. Treat..

[25] Fung S.-C., Fillenz M. (1983). The role of pre-synaptic GABA and benzodiazepine receptors in the control of noradrenaline release in rat hippocampus. Neurosci. Lett..

[26] Schmid G., Chittolini R., Raiteri L., Bonanno G. (1999). Differential effects of zinc on native GABAA receptor function in rat hippocampus and cerebellum. Neurochem. Int..

[27] Barnes R.J., Dhanoa M.S., Lister S.J. (1989). Standard normal variate transformation and de-trending of near-infrared diffuse reflectance spectra. Appl. Spectrosc..

[28] Mok D.K.W., Chau F.T. (2006). Chemical information of Chinese medicines: A challenge to chemist. Chemom. Intell. Lab. Syst..

[29] Bonfiglio T., Olivero G., Vergassola M., Di Cesare Mannelli L., Pacini A., Iannuzzi F., Summa M., Bertorelli R., Feligioni M., Ghelardini C. (2019). Environmental training is beneficial to clinical symptoms and cortical presynaptic defects in mice suffering from experimental autoimmune encephalomyelitis. Neuropharmacology.

[30] Rodriguez A., Zhang H., Klaminder J., Brodin T., Andersson P.L., Andersson M. (2018). ToxTrac: A fast and robust software for tracking organisms. Methods Ecol. Evol..

[31] Bonfiglio T., Olivero G., Merega E., Di Prisco S., Padolecchia C., Grilli M., Milanese M., Di Cesare Mannelli L., Ghelardini C., Bonanno G. (2017). Prophylactic versus therapeutic fingolimod: Restoration of presynaptic defects in mice suffering from experimental autoimmune encephalomyelitis. PLoS ONE.

[32] Grilli M., Pittaluga A., Merlo-Pich E., Marchi M. (2009). NMDA-mediated modulation of dopamine release is modified in rat prefrontal cortex and nucleus accumbens after chronic nicotine treatment. J. Neurochem..

[33] Olivero G., Grilli M., Vergassola M., Bonfiglio T., Padolecchia C., Garrone B., Di Giorgio F.P., Tongiani S., Usai C., Marchi M. (2018). 5-HT2A-mGlu2/3 receptor complex in rat spinal cord glutamatergic nerve endings: A 5-HT2A to mGlu2/3 signalling to amplify presynaptic mechanism of auto-control of glutamate exocytosis. Neuropharmacology.

[34] Hammer Ø., Harper D.A.T., Ryan P.D. (2001). Past: Paleontological statistics software package for education and data analysis. Palaeontol. Electron..

[35] Jolliffe I.T.T. (2002). Principal Component Analysis.

[36] Wold S., Esbensen K., Geladi P. (1987). Principal component analysis. Chemom. Intell. Lab. Syst..

[37] Turrini F., Donno D., Beccaro G.L., Zunin P., Pittaluga A., Boggia R. (2019). Pulsed Ultrasound-Assisted Extraction as an Alternative Method to Conventional Maceration for the Extraction of the Polyphenolic Fraction of Ribes nigrum Buds: A new category of food supplements proposed by the Finnover project. Foods.

[38] Turrini F., Donno D., Boggia R., Beccaro G.L., Zunin P., Leardi R., Pittaluga A.M. (2019). An innovative green extraction and re-use strategy to valorize food supplement by-products: Castanea sativa bud preparations as case study. Food Res. Int..

[39] Boggia R., Turrini F., Anselmo M., Zunin P., Donno D., Beccaro G.L. (2017). Feasibility of UV–VIS–Fluorescence spectroscopy combined with pattern recognition techniques to authenticate a new category of plant food supplements. J. Food Sci. Technol..

[40] Olivero G., Turrini F., Vergassola M., Boggia R., Zunin P., Donno D., Beccaro G.L., Grilli M., Pittaluga A. (2020). The 3Rs: Reduction and refinement through a multivariate statistical analysis approach in a behavioural study to unveil anxiolytic effects of natural extracts of *Tilia tomentosa*. Biomed. Sci. Eng..

[41] Donno D., Boggia R., Zunin P., Cerutti A.K., Guido M., Mellano M.G., Prgomet Z., Beccaro G.L. (2016). Phytochemical fingerprint and chemometrics for natural food preparation pattern recognition: An innovative technique in food supplement quality control. J. Food Sci. Technol..

[42] Leussis M.P., Bolivar V.J. (2006). Habituation in rodents: A review of behavior, neurobiology, and genetics. Neurosci. Biobehav. Rev..

[43] Takeda H., Tsuji M., Matsumiya T. (1998). Changes in head-dipping behavior in the hole-board test reflect the anxiogenic and/or anxiolytic state in mice. Eur. J. Pharm..

[44] Berridge C.W., Dunn A.J. (1989). Restraint-stress-induced changes in exploratory behavior appear to be mediated by norepinephrine-stimulated release of CRF. J. Neurosci..

[45] Rodríguez Echandía E.L., Broitman S.T., Fóscolo M.R. (1987). Effect of the chronic ingestion of chlorimipramine and desipramine on the hole board response to acute stresses in male rats. Pharm. Biochem. Behav..

[46] Wang Y., Kasper L.H. (2014). The role of microbiome in central nervous system disorders. Brain. Behav. Immun..

[47] Masana M.F., Tyrovolas S., Kollia N., Chrysohoou C., Skoumas J., Haro J.M., Tousoulis D., Papageorgiou C., Pitsavos C., Panagiotakos D.B. (2019). Dietary Patterns and Their Association with Anxiety Symptoms among Older Adults: The ATTICA Study. Nutrients.

[48] Head K.A., Kelly G.S. (2009). Nutrients and botanicals for treatment of stress: Adrenal fatigue, neurotransmitter imbalance, anxiety, and restless sleep. Altern. Med. Rev..

[49] Andreescu C., Varon D. (2015). New Research on Anxiety Disorders in the Elderly and an Update on Evidence-Based Treatments. Curr. Psychiatry Rep..

[50] Andreescu C., Lee S. (2020). Anxiety Disorders in the Elderly. Advances in Experimental Medicine and Biology.

[51] Vink D., Aartsen M.J., Schoevers R.A. (2008). Risk factors for anxiety and depression in the elderly: A review. J. Affect. Disord..

[52] Kessler R.C., Birnbaum H.G., Shahly V., Bromet E., Hwang I., McLaughlin K.A., Sampson N., Andrade L.H., de Girolamo G., Demyttenaere K. (2010). Age differences in the prevalence and co-morbidity of DSM-IV major depressive episodes: Results from the WHO World Mental Health Survey Initiative. Depress. Anxiety.

[53] Amico J.A., Mantella R.C., Vollmer R.R., Li X. (2004). Anxiety and stress responses in female oxytocin deficient mice. J. Neuroendocr..

[54] Marques A.A., Bevilaqua M.C.D.N., Da Fonseca A.M.P., Nardi A.E., Thuret S., Dias G.P. (2016). Gender Differences in the Neurobiology of Anxiety: Focus on Adult Hippocampal Neurogenesis. Neural Plast..

[55] Puchol S., Santafe A., Solis M., Sanz V. (2011). Properties of four herbal remedies traditionally used to treat anxiety. Basic Clin. Pharm. Toxicol..

[56] Cavadas C., Fontes Ribeiro C.A., Santos M.S., Cunha A.P., Macedo T., Caramona M.M., Cotrim M.D. (1997). In vitro study of the interaction of *Tilia europeae* L. aqueous extract with GABA(A) receptors in rat brain. Phyther. Res..

[57] Noguerón-Merino M.C., Jiménez-Ferrer E., Román-Ramos R., Zamilpa A., Tortoriello J., Herrera-Ruiz M. (2015). Interactions of a standardized flavonoid fraction from *Tilia americana* with Serotoninergic drugs in elevated plus maze. J. Ethnopharmacol..

[58] Pérez-Ortega G., Guevara-Fefer P., Chávez M., Herrera J., Martínez A.L., González-Trujano M.E. (2008). Sedative and anxiolytic efficacy of *Tilia americana* var. *mexicana* inflorescences used traditionally by communities of State of Michoacan, Mexico. J. Ethnopharmacol..

[59] Aguirre-Hernández E., Martínez A.L.L., González-Trujano M.E.E., Moreno J., Vibrans H., Soto-Hernández M. (2007). Pharmacological evaluation of the anxiolytic and sedative effects of *Tilia americana* L. var. *mexicana* in mice. J. Ethnopharmacol..

[60] Conley M.N., Wong C.P., Duyck K.M., Hord N., Ho E., Sharpton T.J. (2016). Aging and serum MCP-1 are associated with gut microbiome composition in a murine model. PeerJ.

[61] Laukens D., Brinkman B.M., Raes J., De Vos M., Vandenabeele P. (2015). Heterogeneity of the gut microbiome in mice: Guidelines for optimizing experimental design. FEMS Microbiol. Rev..

[62] Vital M., Harkema J.R., Rizzo M., Tiedje J., Brandenberger C. (2015). Alterations of the Murine Gut Microbiome with Age and Allergic Airway Disease. J. Immunol. Res..

[63] Wax T.M., Teena M.W. (1977). Effects of Age, Strain, and Illumination Intensity on Activity and Self-Selection of Light-Dark Schedules in Mice. J. Comp. Physiol. Psychol..

[64] Hodge C.W., Mehmert K.K., Kelley S.P., McMahon T., Haywood A., Olive M.F., Wang D., Sanchez-Perez A.M., Messing R.O. (1999). Supersensitivity to allosteric GABA(A) receptor modulators and alcohol in mice lacking PKCε. Nat. Neurosci..

[65] Quoilin C., Didone V., Tirelli E., Quertemont E. (2010). Ontogeny of the stimulant and sedative effects of ethanol in male and female Swiss mice: Gradual changes from weaning to adulthood. Psychopharmacology.

[66] Yin M., Ikejima K., Wheeler M.D., Bradford B.U., Seabra V., Forman D.T., Sato N., Thurman R.G. (2000). Estrogen is involved in early alcohol-induced liver injury in a rat enteral feeding model. Hepatology.

[67] Iimuro Y., Frankenberg M.V., Arteel G.E., Bradford B.U., Wall C.A., Thurman R.G. (1997). Female rats exhibit greater susceptibility to early alcohol-induced liver injury than males. Am. J. Physiol. Gastrointest. Liver Physiol..

[68] Ikejima K., Enomoto N., Iimuro Y., Ikejima A., Fang D., Xu J., Forman D.T., Brenner D.A., Thurman R.G. (1998). Estrogen increases sensitivity of hepatic Kupffer cells to endotoxin. Am. J. Physiol. Gastrointest. Liver Physiol..

[69] Morgan M.A., Pfaff D.W. (2001). Effects of estrogen on activity and fear-related behaviors in mice. Horm. Behav..

[70] Shansky R.M., Glavis-Bloom C., Lerman D., McRae P., Benson C., Miller K., Cosand L., Horvath T.L., Arnsten A.F.T. (2004). Estrogen mediates sex differences in stress-induced prefrontal cortex dysfunction. Mol. Psychiatry.

[71] Lebron-Milad K., Milad M.R. (2012). Sex differences, gonadal hormones and the fear extinction network: Implications for anxiety disorders. Biol. Mood Anxiety Disord..

[72] Rai A., Gill M., Kinra M., Shetty R., Krishnadas N., Rao C., Sumalatha S., Kumar N. (2019). Catechin ameliorates depressive symptoms in Sprague Dawley rats subjected to chronic unpredictable mild stress by decreasing oxidative stress. Biomed. Rep..

[73] Kalshetti P., Alluri R., Thakurdesai P. (2015). Antidepressant and anti-anxiety effect of ellagic acid from Punica granatum L. rind in olfactory bulbectomy model in rats. Int. J. Pharm. Sci. Rev. Res..

[74] Mansouri M.T., Soltani M., Naghizadeh B., Farbood Y., Mashak A., Sarkaki A. (2014). A possible mechanism for the anxiolytic-like effect of gallic acid in the rat elevated plus maze. Pharm. Biochem. Behav..

[75] Lee B., Sur B., Kwon S., Yeom M., Shim I., Lee H., Hahm D.H. (2013). Chronic administration of catechin decreases depression and anxiety-like behaviors in a rat model using chronic corticosterone injections. Biomol. Ther..

[76] Samad N., Saleem A., Yasmin F., Shehzad M.A. (2018). Quercetin protects against stress-induced anxiety- and depression-like behavior and improves memory in male mice. Physiol. Res..

[77] Bhutada P., Mundhada Y., Bansod K., Ubgade A., Quazi M., Umathe S., Mundhada D. (2010). Reversal by quercetin of corticotrophin releasing factor induced anxiety- and depression-like effect in mice. Prog. Neuro-Psychopharmacol. Biol. Psychiatry.

[78] Wang W., Yang L., Liu T., Wang J., Wen A., Ding Y. (2020). Ellagic acid protects mice against sleep deprivation-induced memory impairment and anxiety by inhibiting TLR4 and activating Nrf2. Aging.

